# Hair testosterone and cortisol interactively predict problematic pornography use in a male sample

**DOI:** 10.1556/2006.2025.00489

**Published:** 2026-03-19

**Authors:** Tom Malte Burkardt, Rudolf Stark, Tobias Stalder, Oliver T. Wolf, Tim Klucken, Matthias Brand, Silke M. Müller

**Affiliations:** 1 Department of General Psychology: Cognition, Faculty of Computer Science, University of Duisburg-Essen, Germany; 2 Department of Psychotherapy and Systems Neuroscience, Justus Liebig University Giessen, Germany; 3 Department of Clinical Psychology and Psychotherapy, University of Siegen, Germany; 4 Department of Cognitive Psychology, Ruhr University Bochum, Germany; 5 Center for Behavioral Addiction Research (CeBAR), Center for Translational Neuro- and Behavioral Sciences (C-TNBS), University Hospital Essen, University of Duisburg-Essen, Germany; 6 Erwin L. Hahn Institute for Magnetic Resonance Imaging, Essen, Germany

**Keywords:** steroid hormones, behavioral addiction, porn addiction, compulsive sexual behavior disorder, dual-hormone hypothesis, impulsivity, risk taking, stress

## Abstract

**Background and aims:**

Steroid hormones, such as testosterone and cortisol, may play a role in addictive behaviors, such as problematic pornography use (PPU). According to the dual-hormone hypothesis, their effects are probably interactive and may influence specific behavioral tendencies, like risk taking and impulsivity, which may, in turn, contribute to PPU. To examine these relationships, the present research utilizes endocrine hair analyses, providing a robust index of long-term hormone secretion.

**Methods:**

The sample (*N* = 252) was part of a multi-center study (FOR2974), including male participants who consume pornography at least occasionally. Testosterone and cortisol concentrations were determined from a proximal 3 cm hair sample via Liquid Chromatography-Tandem Mass Spectrometry. Additionally, decision-making paradigms (i.e., Delay-Discounting Task, Game of Dice Task) and questionnaires assessing impulsivity and symptoms of PPU were used.

**Results:**

Testosterone was positively related to PPU, but showed no significant associations with impulsivity and risk taking in a moderated mediation model. Furthermore, cortisol and testosterone interacted with a significant positive effect of testosterone on PPU severity for participants with low cortisol levels, and a non-significant effect in case of high cortisol levels.

**Discussion and conclusion:**

This study delivers novel evidence for an association between basal testosterone and cortisol levels in PPU for males. However, the assumed association between hormonal levels, risk taking and impulsivity could not be supported.

## Introduction

Using pornography constitutes a highly popular leisure time activity in the current age (Wright, Tokunaga, & Herbenick, 2023). Pornography usage is not dangerous or harmful per se for most people (Binnie & Reavey, 2020a). Some studies furthermore report positive effects of pornography usage, such as improved attitudes towards sex and the opposing gender (Hesse & Pedersen, 2017) or educational effects (McCormack & Wignall, 2017). However, an estimated 3.2% of users develop a problematic usage (Bőthe et al., 2024), which can present itself in the repeated experience of negative consequences due to their pornography consumption and repeated unsuccessful attempts to reduce the behavior (Binnie & Reavey, 2020b). Problematic pornography usage (PPU) is especially prevalent in younger men (Lewczuk, Wójcik, & Gola, 2022) and furthermore associated with psychopathological distress (Mennig, Tennie, & Barke, 2022). Whether and how PPU can be defined as a clinical entity remains a subject of discussion (Antons & Brand, 2021). While other representations of problematic online behaviors like gaming disorder have officially been classified in the latest edition of the International Classification of Diseases (ICD-11), PPU is missing a dedicated classification (World Health Organization, 2018). For now, it only receives mention as a potential manifestation of compulsive sexual behavior disorder (CSBD; 6C72). Despite the differing categorization of potentially problematic online behaviors, there might be an underlying common denominator in similar mechanisms being involved in the development and maintenance of the respective problematic/addictive behavior that might be considered within the ICD-11 distinction “other specified disorders due to addictive behaviors” (Brand et al., 2020). A theoretical framework often applied in explaining addictive behaviors is the Interaction of Person-Affect-Cognition-Execution (I-PACE) model (Brand et al., 2019). The model postulates interactions between predisposing, affective, cognitive, and executive variables during the development and maintenance of addictive behaviors, including PPU. In accordance with other addiction theories (e.g., Goldstein & Volkow, 2002; Koob & Volkow, 2010; Robinson & Berridge, 1993), the I-PACE model assumes that repetitive exposure to specific rewards alters the functioning of the brains reward system which, over time, results in sensitization towards addiction-related stimuli causing cue-reactivity and craving as well as attentional biases towards such stimuli. Reinforcement learning and intensification processes promote execution of the addictive behavior even if the behavior itself is no longer perceived as gratifying. The I-PACE model assumes specific predisposing variables and vulnerability factors, including temperamental features and genetics, to interact with internal affective and cognitive mechanisms. One part of predisposing variables which is quite understudied hitherto, is hormonal influences.

This study aims to draw connections between testosterone and PPU. This connection might be facilitated via associations with impulsivity and risk taking. Furthermore, interactions between testosterone and cortisol are hypothesized. In the following, we will address empirical findings on the theoretically assumed connections.

### The link between testosterone and PPU

With (problematic) pornography use being vastly male-dominated (Borgogna, Griffin, Grubbs, & Kraus, 2022), the question arises whether biological differences such as different compositions of steroid hormones (e.g., testosterone) could partly amount for the continued use. Indirect support for this notion comes from evidence linking higher testosterone levels to an increased risk for alcohol use disorder, especially in males (Erol, Ho, Winham, & Karpyak, 2019; Li, Ramoz, Derrington, & Dreher, 2020). While findings for substance use disorders are not directly applicable to non-substance related issues, such as PPU, there still appears to be some overlap (Grant, Potenza, Weinstein, & Gorelick, 2010). Empirical findings on the role of testosterone in PPU and other non-substance related addictions are scarce and mixed: Associations have been reported for hypersexuality (Chatzittofis et al., 2020) and CSBD (Ballester-Arnal, Castro-Calvo, García-Barba, Nebot-García, & Gil-Llario, 2023; Rodríguez-Nieto, Dewitte, Sack, & Schuhmann, 2021). Interestingly, both studies regarding CSBD showed an overall positive association of testosterone and CSBD symptoms, but no significant association with sexual behavior such as frequency of masturbation or pornography use. This suggests that the role of testosterone on CSBD, and potentially PPU, might be more complex than testosterone solely being a driver for sexual activity. Looking at other behavioral addictions, no significant difference in testosterone was found between young men with and without gaming disorder (Koenig, Thaler, Parzer, Resch, & Kaess, 2019) and between individuals with gambling disorder and control participants (Blanco, Ibáñez, Blanco-Jerez, Baca-Garcia, & Sáiz-Ruiz, 2001). With existing results in comparative fields being few and mixed, the question remains whether and how testosterone may play a role in PPU.

### Risk taking and impulsivity are associated with testosterone

In this line, it might be important to consider links between testosterone and factors which are not inherently sexually connotated, like risk taking, as proposed by Rodríguez-Nieto et al. (2021). Testosterone and risk taking may be connected from an evolutionary standpoint, as testosterone is associated with competition, e.g., the acquisition of resources and status (van Anders, Goldey, & Kuo, 2011), which can theoretically be achieved by taking risks (French, Kamil, Leger, & Daly, 2001). Several works support this relation between testosterone and risk taking (Apicella, Carré, & Dreber, 2015; Evans & Hampson, 2014; Kurath & Mata, 2018; Stanton, Liening, & Schultheiss, 2011), although it should be noted that other studies show conflicting results (Derntl, Pintzinger, Kryspin-Exner, & Schöpf, 2014; Ronay, van der Meij, Oostrom, & Pollet, 2018; Sapienza, Zingales, & Maestripieri, 2009; Stanton, Welker, Bonin, Goldfarb, & Carré, 2021). Relatedly, impulsivity also shows associations with levels of testosterone (Borráz-León, Nickels McLean, Jurgensen, & Maestripieri, 2023; Kurath & Mata, 2018; Wu et al., 2020) and plays a major role in addictive processes (Lee, Hoppenbrouwers, & Franken, 2019). While risk taking and impulsivity share certain features, both constructs still describe mechanisms distinct from each other (Nigg, 2017), making it worth to look at both factors separately.

### Interplay between cortisol and testosterone

Adding on to the testosterone-competition association described by van Anders et al. (2011), competition may further be dependent on the relation between testosterone and cortisol (Casto & Edwards, 2016). Subsequently, the relationship between testosterone, risk taking and impulsivity may also be dependent on adrenal activity. This would be in line with the Dual Hormone Hypothesis (DHH; Mehta & Josephs, 2010), which proposes an influence of testosterone on dominance behavior, but only in people who are low in cortisol (Mehta, Welker, Zilioli, & Carré, 2015). The DHH has also been applied more broadly to constructs like risk taking and impulsivity (Kurath & Mata, 2018). This connection with cortisol is proposed as a result of an interplay between the hypothalamic-pituitary-gonadal (HPG-) and the hypothalamic-pituitary-adrenal (HPA-) axes (Mehta & Josephs, 2010). Accordingly, cortisol, released by the HPA axis, interferes with the function of the HPG axis, which releases testosterone (Johnson, Kamilaris, Chrousos, & Gold, 1992). As a result, testosterone-affected behavior is potentially dampened. Effects of testosterone on risk taking are therefore hypothesized to be diminished or even reversed by high concentrations of cortisol (Mehta et al., 2015). Both axes are further hypothesized to play a role in CSBD (Chatzittofis et al., 2022), with findings hinting at a hyperactivity of the HPA axis (Chatzittofis et al., 2016), and a slight hypoactivity of the HGP axis (Chatzittofis et al., 2020) in hypersexual men which warrants study approaches assessing activity of both axes in conjunction. Combining these theoretical assumptions, this study argues that possible testosterone-associations with risk taking and impulsivity, may be moderated by cortisol levels.

Risk taking and impulsivity can conclusively also be of relevance for PPU, given that a recent large scale study reported more disadvantageous (i.e., risky) decision making and higher levels of impulsivity, in individuals with pathological internet use (including PPU) compared to individuals with nonproblematic use (Müller et al., 2025). Looking at specific findings for PPU, impulsivity seems to be a consistently associated factor, according to a recent review (Gaudet et al., 2025). Studies on risk taking in PPU/CBSD report mixed findings with some studies providing support for a positive association with risk preferences measured via different decision-making paradigms (Mulhauser et al., 2014; Wang, Qu, Li, Tang, & Li, 2024) while other studies reported null findings (Engel et al., 2025; Müller & Antons, 2023). More studies are therefore needed to clarify the relation between risk taking and PPU.

### Methodological advances of endocrine assessments via hair

Most hormone research in the addiction context is based upon single-time saliva or blood sampling, which gives insights into the current hormonal profile, but lacks robustness against potential fluctuations, like the diurnal rhythm for example in the case of testosterone (Diver, Imtiaz, Ahmad, Vora, & Fraser, 2003). It is furthermore of interest to assess hormonal levels in a way that allows to infer hormonal information for longer time periods, especially if hormonal values are to be correlated to relatively stable traits (e.g., impulsivity) as argued by Dabbs (1990). This study therefore assesses hormones in hair which offer a practical and non-invasive alternative to assessments in blood, saliva or urine (Stalder & Kirschbaum, 2012). Hair samples are thought to provide an index of cumulative hormone exposure over several months (Short et al., 2016). Given their long-term nature, hair-derived analytes have been shown to be intra-individually stable (Stalder et al., 2012) and relatively robust to a range of potential covariates (Stalder et al., 2017) and acute influences (Grass et al., 2015).

### The present study

This study aims to explore hormonal associations (measured via hair samples) with risk taking, impulsivity and their significance in explaining PPU symptoms. Based on research in related fields of behavioral addictions and CSBD we hypothesize a positive association between testosterone and PPU. We furthermore expect a mediation effect of this association via risk taking and impulsivity. Drawing on neuroendocrinological theory, we lastly hypothesize an interaction effect between testosterone and cortisol on risk taking and impulsivity.

## Methods

### Participants and procedure

The study is part of a multi-center study of the research unit FOR2974 funded by the German research foundation, which assesses participants across different forms of potentially addictive online behaviors (Brand et al., 2021). As part of the first FOR2974 cohort, the participants are a subsample of the sample reported in Müller et al. (2025). The present hair endocrine data have not been reported previously. This study included all male participants of the cohort, who used pornography at least occasionally in the past twelve months. Although females were recruited, substantial exclusion resulted from use of hormonal contraception use or missing information in regards to it (*n* = 236), missing or non-detectable values in testosterone (*n* = 21), and not using pornography during the last 12 months (*n* = 13). The number of remaining females who met all inclusion criteria (*n* = 2) was deemed too little to include in the analysis. Male participants who were classified as pathological based on a structured interview for Internet-related disorders in one of the behaviors apart from pornography (*n* = 32), were excluded to avoid possible cofounding effects. One Participant with missing values in hypothesis-relevant variables was also excluded. A total of 252 male participants were included in the final data analysis. Participants were between 18 and 42 years of age (*M* = 25.94, *SD* = 6.31) and mainly consisted of students from Germany (for descriptive statistics & further demographics see Table 1).

**Table 1.** Descriptive Statistics

Variable	Range	*M*	*SD*	*N* (%)
Age (years)	18–42	25.94	6.31	–
Relationship status: Single	–	–	–	110 (43.6%)
Relationship status: In a relationship	–	–	–	142 (56.3%)
Occupation: Student	–	–	–	189 (75%)
Occupation: Full-time employed	–	–	–	30 (11.9%)
Occupation: Part-time employed	–	–	–	12 (4.8%)
Occupation: Other	–	–	–	21 (8.3%)
Country of birth: Germany	–	–	–	236 (93.7%)
Country of birth: Other	–	–	–	16 (6.3%)
Severe cases of PPU	–	–	–	11 (4.4%)
Testosterone (pg/mg, Log_10_ +1)	0.05–2.08	0.98	0.32	–
Cortisol (pg/mg, Log_10_ +1)	0.26–2.46	1.47	0.35	–
ACSID-11-PPU-Frequency (mean)	0–3.00	0.67	0.70	–
ACSID-11-PPU-Intensity (mean)	0–2.91	0.64	0.70	–
GDT (net score)	−18–18	11.45	9.09	–
DDT (Log(k))	−9.12–0.34	−6.25	1.51	–
BIS-15 (sum)	18–54	32.79	6.50	–

*Notes.* ACSID-11 = Assessment of Criteria for Specific Internet-use Disorders; GDT = Game of dice Task; DDT = Delay-Discounting Task; BIS-15 = Baratt-Impulsiveness-Scale; Severe cases refer to proposed cut off by Oelker, Rumpf, Brand, and Müller (2024).

The overall study procedure of the research unit FOR2974 was pre-registered at Open Science Framework (doi: 10.17605/OSF.IO/N5CD7). Participants were recruited at multiple sites in Germany from October 2021 until August 2024 via mailing lists, social media, and local advertisements at the investigating sites. After a telephone screening, individuals eligible to participate were invited to take part in the laboratory study which consisted of various computerized tasks, screenings, and questionnaires, as well as the voluntary submission of a hair sample.

### Measures

#### Hormonal assay

Long-term hormone secretion was assessed by hair sampling (conducted at the end of each session). Two hair strands with an approximate diameter of 3 mm were cut as close to scalp as possible from an occipital position at the back of the head, as defined by Huthsteiner, Finke, Peters, Kleinke, et al. (2025). Hair samples were stored wrapped in aluminum foil at each study site until being sent to Dresden LABservice GmbH for analysis. On average, samples were stored for 334 days before being analysed (*M* = 333.51, *SD* = 148.52, Range: 28–866). Endocrine analyses were conducted on 7.5 mg of hair using a previously published Liquid Chromatography-Tandem Mass Spectrometry protocol (Gao et al., 2013). The first proximal 3-cm hair segment was used for analyses to reflect the past three-month-period, based on an average hair growth of approximately 1 cm/month (Wennig, 2000).

#### Behavioral measures

The Game of Dice Task (GDT; Brand et al., 2005) was used to assess risk taking. Participants are instructed to choose a dice number (i.e., 1–6) or a combination of two, three or four numbers which are connected to a sum being possibly won (in case of the number appearing) or lost (in case of the opposite). Afterwards, a virtual dice is rolled and the resulting loss or win gets added to the players' balance. Choosing a single dice number is the riskiest decision, because of a low likelihood of choosing the correct number, and conjunctly higher potential wins but also (more likely) losses. Conversely, choosing a combination of several numbers increases the likelihood of being right, but also decreases the potentially won sum. After 18 trials, the paradigm is terminated and the number of disadvantageous decisions (i.e., choosing a single number or a combination of two) is subtracted from the number of advantageous ones (i.e., choosing a combination of three or four numbers), representing the net score. Lower scores represent a preference for choices accompanied by more risk.

In order to measure impulsive choice tendencies, a short Delay-Discounting Task (DDT) was used (Koffarnus & Bickel, 2014). In this task, participants make five fictional choices between receiving 500€ now or 1000€ delayed (e.g., two weeks). The presented time periods are varied per choice and adjusted according to the choices made previously. The final score consists of a logarithmized discount rate *k*. Higher values indicate a preference for immediate but smaller rewards. In both tasks, no real money was given to the participants.

#### Questionnaires

The main outcome variable of this study, namely PPU symptom severity, was measured by using the Assessment of Criteria for Specific Internet-Use Disorders (ACSID-11; Müller et al., 2022) which is a screening tool for assessing the severity of different forms of problematic usage of the internet. The scale consists of 11 questions representing ICD-11 criteria for gaming disorder and (in an integrated response format) also for other types of behaviors, including pornography use. For each behavior, responses are given on two scales which assess the frequency and intensity for each symptom (e.g. “In the past 12 months, have you given [the activity] an increasingly higher priority than other activities or interests in your daily life?”) and were answered on four-point Likert scales (frequency: from 0 - “never” to 3 - “often”; intensity: from 0 - “not intense” to 3 - “intense”). Regarding PPU, both ACSID-11 subscales showed excellent internal consistency (PPU-Frequency: Cronbachs *α* = 0.94; PPU-Intensity: Cronbachs *α* = 0.93).

The German short version of the Barratt Impulsiveness Scale (BIS-15; Meule, Vögele, & Kübler, 2011) was used to assess trait impulsivity, via 15 items. Items were answered on a four-point Likert scale ranging from 1 – “rarely/never” to 4 – “almost always/always”. Higher scores indicate a higher propensity for impulsivity. The scale showed good internal consistency (Cronbachs *α* = 0.80).

### Statistical analysis

**Fig. 1.**
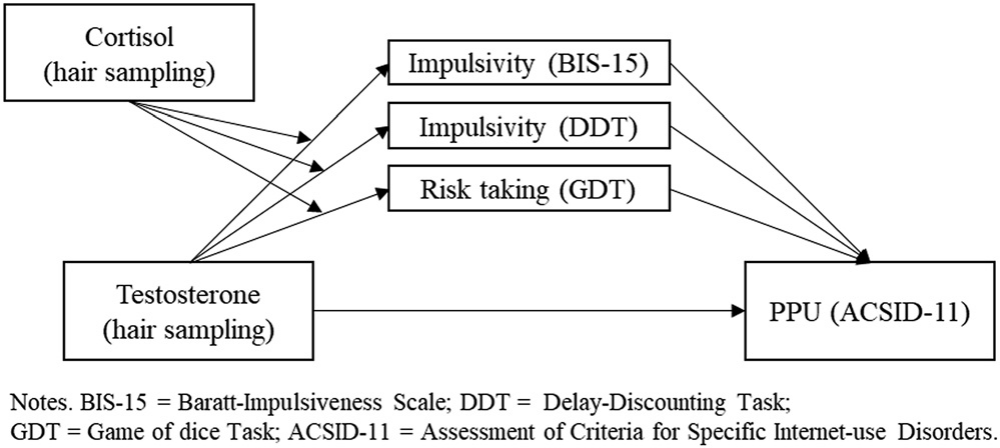
Proposed moderated mediation model

All analyses were carried out using IBM SPSS Statistics version 29. The hormonal data was tested for skewness and subsequently transformed (i.e., Log10(x)), furthermore a constant (+1) was added to the values to avoid negative values. Using a Boxplot analysis identified three extreme outliers (Q3 + 3 × IQR) regarding testosterone (2) or cortisol (1) values, which were excluded. Pearson correlations were used for bivariate correlational analyses. To test the hypothesized assumptions, a moderated mediation model (Fig. 1) was calculated, using the PROCESS extension (Hayes, 2013) with the corresponding model (i.e., model 7). All regression analyses were tested for multicollinearity and heteroscedasticity. The heteroscedasticity-robust standard error HC3 was used to correct for violations of the assumption (Long & Ervin, 2000). In order to account for potential influences of storage duration of hair samples (Huthsteiner, Finke, Peters, Klucken, & Stalder, 2025), the results of regression models were tested for robustness to the inclusion of the variable as a covariate.

### Ethics

The study procedures were carried out in accordance with the Declaration of Helsinki. The responsible ethics committee at each recruitment site approved the study. The overall study procedure was approved by the local ethics committee of the University of Duisburg-Essen (ID: 1911APBM0457). All participants were thoroughly informed about the study prior to partaking and gave informed consent.

## Results

Table 1 shows descriptive statistics for demographic and hypotheses-relevant variables.

### Correlational analysis

As seen in Table 2, testosterone correlated with both ACSID-11 subscales, showing small positive effects, but not with the proposed mediators (performance on the DDT, GDT, and BIS-15 scores). Based on the correlations, the proposed moderated mediation model was calculated with the intensity scale of the ACSID-11 as dependent variable due to missing correlations between the frequency scale and the mediator variables.

**Table 2.** Pearson correlations for all hypotheses-relevant variables

	1	2	3	4	5	6
1. Testosterone	–					
2. Cortisol	.311**	–				
3. ACSID-11-PPU-Frequency	.215**	−.044	–			
4. ACSID-11-PPU-Intensity	.202**	−.029	.885**	–		
5. GDT	−.007	.127*	−.123	−.140*	–	
6. DDT	−.023	−.026	.103	.137*	−.059	–
7. BIS-15	.095	.042	.213**	.186**	−.093	.034

*Notes. N* = 252, *p < .05, **p < .01. ACSID-11 = Assessment of Criteria for Specific Internet-use Disorders; GDT = Game of Dice Task; DDT = Delay-Discounting Task; BIS-15 = Baratt-Impulsiveness-Scale.

### Moderated mediation model

The correlational analysis and the VIF-values (<1.2 for all variables) did not indicate multicollinearity. The heteroscedasticity-robust estimator (HC3) was used to test the proposed model (see Fig. 1) with age as a covariate. Table 3 shows the moderation effects of testosterone-cortisol interactions as well as the direct effects of testosterone and the mediator variables on PPU symptom severity. The inclusion of all predictors explained 10.1% of the variance (see Table 3). Testosterone had no significant effect on the mediator variables of GDT, DDT or BIS-15, but significantly predicted PPU symptom severity (measured by the ACSID-11-PPU-Intensity score) with a small effect. Cortisol did not significantly moderate any path of testosterone on the mediators. Out of the mediator variables, only the BIS-15 score had a significant, albeit small effect on the dependent variable. Given that testosterone did not show significant effects on the mediator variables with and without the inclusion of cortisol as a moderator, the assumed mediation effects can be dismissed. Furthermore, the results remained largely similar if storage duration of hair samples was included as a covariate. All specific mediation results of the model (Fig. 1) are presented in the supplementary material.

**Table 3.** Moderation effects of testosterone-cortisol interactions on impulsivity and risk taking

Outcome	Predictors	*β*	*SE*	*p*	95% CI
GDT	Testosterone	−0.036	0.074	.628	[−0.18, 0.11]
	Cortisol	0.142	0.077	.064	[−0.01, 0.29]
	Testosterone x Cortisol	0.001	0.060	.986	[−0.12, 0.20]
*R*^2^ = 0.028, *F* = 2.079, *p* = .084					
DDT	Testosterone	0.012	0.073	.873	[−0.13, 0.16]
	Cortisol	−0.015	0.079	.854	[−0.17, 0.14]
	Testosterone x Cortisol	0.110	0.078	.163	[−0.05, 0.26]
*R*^2^ = 0.042, *F* = 1.706, *p* = .149					
BIS-15	Testosterone	0.088	0.070	.205	[−0.05, 0.23]
	Cortisol	0.015	0.059	.796	[−0.10, 0.13]
	Testosterone x Cortisol	0.028	0.056	.617	[−0.08, 0.14]
*R*^2^ = 0.010, *F* = 0.687, *p* = .601					
ACSID-11-PPU-Intensity	Testosterone	0.196	0.068	.004	[0.06, 0.33]
	GDT	−0.121	0.073	.098	[−0.27, 0.02]
	DDT	0.122	0.072	.090	[−0.02, 0.26]
	BIS-15	0.153	0.059	.010	[0.04, 0.27]
*R*^2^ = 0.103, *F* = 5.317, *p* < .001					

*Notes.* ACSID-11 = Assessment of Criteria for Specific Internet-use Disorders; GDT = Game of Dice Task; DDT = Delay-Discounting Task; BIS-15 = Baratt-Impulsiveness-Scale.

### Post-hoc analysis: testosterone-cortisol interaction on PPU severity

**Fig. 2.**
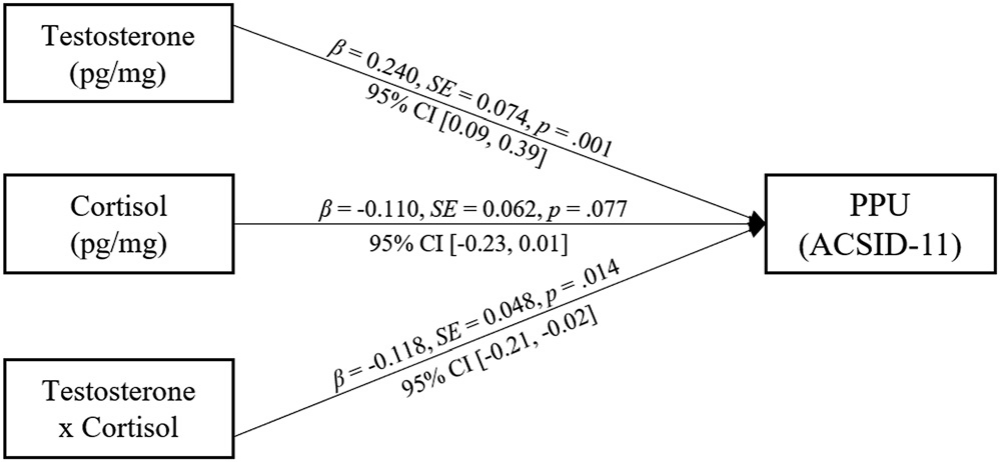
Moderation analysis of testosterone, cortisol, and their interaction on PPU symptom severity (ACSID-11-PPU-Intensity).

Based upon the assumption that effects which are offset by testosterone are moderated by cortisol, the interaction effect between testosterone and cortisol levels on PPU symptom severity was tested. Therefore, a standard moderation model (i.e., PROCESS model 1) was applied using the heteroscedasticity-robust estimator (HC3) with the ACSID-11-PPU-Intensity score as the dependent variable and age as a covariate. The model significantly explained 6.7% of variance (*R*^2^ = 0.067, *F* = 4.518, *p* = .002). As illustrated in Fig. 2, cortisol significantly moderated the effect of testosterone on PPU symptom severity (Δ*R*^2^ = .014, *F*(1, 247) = 6.125, *p* = .014).

The effects were similar when using the frequency scale of the ACSID-11 as the criterion in the model. Results also remained robust to the inclusion of storage duration of the hair samples as a covariate. In additional analyses, we aimed to test whether the variables assumed as mediators (GDT, DDT, BIS-15) may instead function as additional moderators within separate moderated regression models. None of the three-way interactions were significant.

**Fig. 3.**
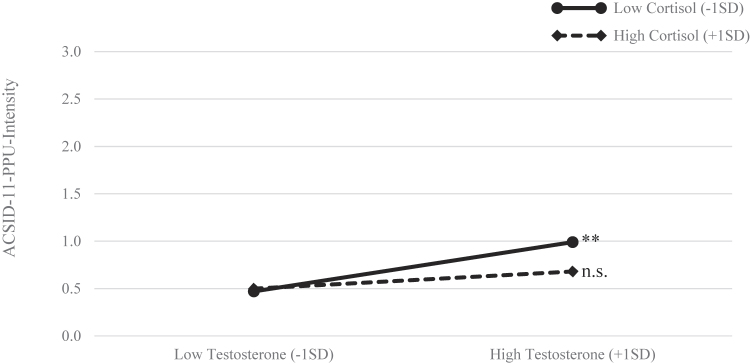
Simple slope analysis of the interaction between cortisol and testosterone on PPU symptom severity

To further investigate the moderating effect of cortisol, a simple slope analysis was performed based on the unstandardized coefficients (Fig. 3). The analysis showed the effect of testosterone on PPU symptom severity to be the most pronounced in participants with low levels of cortisol (*B* = 0.78, *p* < .001). The effect is lower and non-significant in participants with high levels of cortisol (*B* = 0.26, *p* = .180).

## Discussion

This is the first study demonstrating associations of basal long-term testosterone and cortisol levels with PPU. We assumed testosterone to be related to PPU, mediated by impulsivity and risk taking, and cortisol to moderate this testosterone effect. Testosterone positively predicted PPU, but was not related to impulsivity or risk taking, with and without cortisol as a moderator. However, testosterone interacted with cortisol regarding PPU, showing that the association is strongest in individuals with low cortisol levels.

The result that testosterone is positively associated with PPU is consistent with previous studies linking testosterone to symptoms of CSBD (Ballester-Arnal et al., 2023; Rodríguez-Nieto et al., 2021) or hypersexuality (Chatzittofis et al., 2020), underlining the overlap between these concepts and PPU. Our study further enriches current evidence on the relationships between hormones and PPU/CSBD by using hair testosterone analysis, whereas previous studies used single-time blood or saliva sampling. Studies on other forms of addictive behaviors (Blanco et al., 2001; Koenig et al., 2019) did not find the testosterone-symptoms relationship, which indicates that heightened testosterone may be a behavior-specific predisposition for PPU in regard to the I-PACE model (Brand et al., 2019). Given that the interaction between cortisol and testosterone was also significant, the potentially predisposing effect for PPU could be specified for males who are low in cortisol and high in testosterone.

Furthermore, pornography usage and sexual activity could in turn affect hormonal levels, which was not addressed in this study. Longitudinal data by Štulhofer, Koletić, Landripet, Hald, and Ćuća (2019) reported no relation between frequency of masturbation or pornography consumption and testosterone, which is in line with findings from Ballester-Arnal et al. (2023). However, Goldey und van Anders (2015) argue that the contextualization of a sexual stimulus matters for changes in testosterone levels. According to the authors, nurturing contexts may decrease testosterone, while competitive contextualization increases it. Pornography consumption does not clearly fit either context and its contextualization may differ between users and use situations. Therefore, a potential bidirectionality between pornography use, accompanied sexual activity and testosterone needs further exploration.

The testosterone-cortisol interaction effect may be considered partly confirming the DHH, i.e., that significant effects of testosterone on PPU are influenced by cortisol. However, the initial hypothesis that this interaction would affect impulsivity and risk taking, and that testosterone is linked to both, was not confirmed. This finding contradicts a recent review, stating that testosterone is associated with risk taking and impulsivity (Raji, Dinesh, & Sharma, 2025). Previous studies including hair hormone analyses and risk taking are limited and inconclusive, with a null finding regarding testosterone but a significant interaction with cortisol (Ronay et al., 2018) and another study showing no interaction of testosterone and cortisol on risk taking (Shields, Ivory, & Telzer, 2019). The missing associations in our study could be explained by the assessment of risk taking via the GDT, as most previous studies that found effects of testosterone on risk taking used the Balloon Analogue Risk Task (Mehta et al., 2015; Peper, Koolschijn, Cédric, & Crone, 2013) or the Iowa Gambling Task (Evans & Hampson, 2014; Stanton et al., 2011). Both tasks assess risk taking under ambiguous conditions, where the participant is, at least initially, unaware of probabilities and potential outcomes, and has to learn the contingencies and rules of winning/losing, based on feedback. Hormonal influences concerning risk taking might therefore be more relevant in situations where decision making under ambiguity is required. The missing hormonal interactions regarding impulsivity and risk taking could be furthermore attributed to the form of hormonal assessment. Although there are a few studies providing support of the DHH in hair hormones (Grotzinger et al., 2018; Ronay et al., 2018), most studies testing the DHH predominantly used blood or saliva-sampling (Kurath & Mata, 2018) usually coupled with immunoassay analysis (Dekkers et al., 2019). The authors of the latter meta-analysis therefore argued that current evidence for the DHH might be limited by measurement errors. Relatedly, Knight, Sarkar, Prasad, and Mehta (2020) recommend the Liquid Chromatography-Tandem Mass Spectrometry-analysis of hormone concentrations (as done in our study) for future studies in the context of the DHH. More studies testing the DHH using hormone measures in hair are therefore needed to elucidate whether long-term hormone secretion interact in predicting risk taking and impulsivity. To conclude, our study provides no support for the assumptions of the DHH regarding risk taking and impulsivity, but supports the interaction between testosterone and cortisol in relation to PPU symptoms.

Looking at risk taking and impulsivity in the context of PPU, this study corroborated earlier research regarding positive associations with impulsivity (Gaudet et al., 2025). However, delay discounting did not have a significant effect in our study, conversely to the finding of Antons et al. (2019), which might be due to differing forms of assessment. Overall, these findings underline that impulsivity seems to be associated with PPU in general, but facets of impulsivity potentially differ in their significance in this context (Bocci Benucci, Di Gesto, Ghinassi, Casale, & Fioravanti, 2024). Risk taking was correlated with PPU symptom severity (intensity score) on a bivariate level, but with a small effect size and did not have its own incremental validity in predicting PPU when other variables were entered in the regression. Our current study could therefore not convincingly support the hypothesis that risky decision making is associated with PPU, which is in line with previous studies also using the GDT (Engel et al., 2025) or other forms of assessment (Müller & Antons, 2023). This lack of association may be due to the potentially less risky nature of pornography use compared to non-digital sexual activities (e.g., seeking out sexual activity with a potential partner in real life). For example, the experience of rejection is unlikely when consuming pornography, subsequently deeming it the “safer” choice in comparison to offline sexual activity, which is potentially more attractive to risk-averse individuals. Nonetheless, consuming pornography can still involve social or relational risks, as stigmatization (Macdowall et al., 2025) or conflicts with other people might be the consequence (Kor et al., 2014).

While there was no support for a moderated or direct effect of testosterone on risk taking in this study, cortisol had a close to significant positive direct effect on GDT performance in the moderated mediation model. This finding warrants further investigations of cortisol as a potentially protective factor for risk taking, as this effect was also shown in another study assessing hormones in hair and risk taking (Shields et al., 2019).

### Limitations and future directions

The results of this study are based on cross-sectional data, which limits the ability to draw causal conclusions. For example, the association between testosterone levels and PPU could theoretically be bidirectional. While we did find significant novel associations between hormones and PPU, effect sizes were generally small. Accordingly, the practical implications of the present findings should be interpreted with caution.

This study could not address potential covariates for hormonal values detected in the hair, such as sun exposure (Wester, van der Wulp, Koper, Rijke, & van Rossum, 2016) or washing frequency (Behnke et al., 2021), for which we lacked information for a large part of the sample. However, we consider it unlikely that this has had a strong influence on the present results given that meta-analytic evidence indicates only small effect sizes for associations between hair cortisol and such covariates (Stalder et al., 2017). Furthermore, hair testosterone levels of male participants seem to be unrelated to various sorts of hair treatment, but hair color may have an effect (Johnson-Ferguson et al., 2023).

Another limitation of our study is that we only included male participants and therefore the generalizability of the results is limited. The results should be viewed with caution in terms of their applicability for female or gender-diverse populations, since production of testosterone and the sensitivity to it, varies greatly between biological genders, leading to potentially different effects in sexuality and behavior (Bancroft, 2005). This gender disparity may also be considered in the context of addiction-relevant effects of cortisol (Franco, Paris, Wulfert, & Frye, 2010) and the interaction between testosterone and cortisol (Barel, Shahrabani, & Tzischinsky, 2017). Future studies should therefore investigate non-male populations to clarify whether testosterone, and its interaction with cortisol, is a gender-transcending factor, or posits an exclusively male predisposition. Related variables like sexual orientation, gender identity, or other social factors relating to gender may also affect testosterone (van Anders, Steiger, & Goldey, 2015) and were not assessed in this study. Respective assessments will be obtained in the second cohort of the FOR2974, enabling us to address gender-endocrine relationships more precisely in future studies.

Lastly, the sample was rather young and close to the age group where testosterone secretion usually peaks (Handelsman, Sikaris, & Ly, 2016). As testosterone levels are known to decline with increasing age (Harman, Metter, Tobin, Pearson, & Blackman, 2001), future research seeking to replicate the present results in older age groups would be useful.

### Conclusions

The results of the study offer novel implications for endocrinological effects in PPU using hair sampling as an index of long-term hormone secretion. Elevated testosterone levels in conjunction with low cortisol levels may be associated with PPU in males. However, the mechanisms facilitating this connection are yet to be discovered, as risk taking and impulsivity did not seem to be relevant in this context.

## Supplementary material
