# Supplementary material for: Hair testosterone and cortisol interactively predict problematic pornography use in a male sample
Source: J Behav Addict. 2026 Mar 19;15(1):459–70. doi: 10.1556/2006.2025.00489 (PMC13132414; doi:10.1556/2006.2025.00489)
Supplement: Supplementary file 1 [file jba-15-459-s001.pdf]

Run MATRIX procedure:

***************** PROCESS Procedure for SPSS Version 4.2 *****************

Written by Andrew F. Hayes, Ph.D.   www.afhayes.com
Documentation available in Hayes (2022). www.guilford.com/p/hayes3

****
Model : 7
Y : PPU#1
X : T
M1 : GDT
M2 : DDT
M3 : BIS15
W : C

Covariates:
Age

Sample
Size: 252

****
OUTCOME VARIABLE:
GDT

Model Summary

R	R-sq	MSE	F(HC3)	df1	df2	p
,166	,028	,988	2,079	4,000	247,000	,084

Model

	coeff	se(HC3)	t	p	LLCI	ULCI
constant	,000	,066	-,005	,996	-,130	,129
T	-,036	,074	-,486	,628	-,181	,109
C	,142	,077	1,862	,064	-,008	,293
Int_1	,001	,060	,017	,986	-,118	,120
Age	,096	,054	1,783	,076	-,010	,203

Product terms key:
Int_1   :   T   x   C

Test(s) of highest order unconditional interaction(s):

	R2-chng	F(HC3)	df1	df2	p
X*W	,000	,000	1,000	247,000	,986

****
OUTCOME VARIABLE:
DDT

Model Summary

R	R-sq	MSE	F(HC3)	df1	df2	p
,204	,042	,974	1,706	4,000	247,000	,149

Model

	coeff	se(HC3)	t	p	LLCI	ULCI
constant	-,034	,063	-,541	,589	-,158	,090
T	,012	,073	,160	,873	-,132	,155

C	-,015	,079	-,184	,854	-,170	,141
Int_1	,110	,078	1,400	,163	-,045	,264
Age	,145	,068	2,115	,035	,010	,279

Product terms key:
Int_1 : T x C

Test(s) of highest order unconditional interaction(s):

	R2-chng	F(HC3)	df1	df2	p
X*W	,012	1,959	1,000	247,000	,163

****
OUTCOME VARIABLE:
BIS15

Model Summary

R	R-sq	MSE	F(HC3)	df1	df2	p
,102	,010	1,006	,687	4,000	247,000	,601

Model

	coeff	se(HC3)	t	p	LLCI	ULCI
constant	-,009	,066	-,133	,895	-,138	,121
T	,088	,070	1,270	,205	-,049	,225
C	,015	,059	,259	,796	-,100	,131
Int_1	,028	,056	,500	,617	-,083	,139
Age	-,028	,066	-,423	,672	-,159	,103

Product terms key:
Int_1 : T x C

Test(s) of highest order unconditional interaction(s):

	R2-chng	F(HC3)	df1	df2	p
X*W	,001	,250	1,000	247,000	,617

****
OUTCOME VARIABLE:
PPU#1

Model Summary

R	R-sq	MSE	F(HC3)	df1	df2	p
,320	,103	,916	5,317	5,000	246,000	,000

Model

	coeff	se(HC3)	t	p	LLCI	ULCI
constant	,000	,061	,000	1,000	-,121	,121
T	,196	,068	2,879	,004	,062	,330
GDT	-,121	,073	-1,663	,098	-,265	,022
DDT	,122	,072	1,701	,090	-,019	,263
BIS15	,153	,059	2,603	,010	,037	,269
Age	,043	,065	,662	,509	-,085	,171

****************** DIRECT AND INDIRECT EFFECTS OF X ON Y *****************

Direct effect of X on Y

Effect	se(HC3)	t	p	LLCI	ULCI
,196	,068	2,879	,004	,062	,330

Conditional indirect effects of X on Y:

INDIRECT EFFECT:
T -> GDT -> PPU#1

C	Effect	BootSE	BootLLCI	BootULCI
-,800	,004	,014	-,016	,040
-,109	,004	,011	-,012	,034
,951	,004	,014	-,016	,039

Index of moderated mediation:

	Index	BootSE	BootLLCI	BootULCI
C	,000	,009	-,019	,018

INDIRECT EFFECT:
T -> DDT -> PPU#1

C	Effect	BootSE	BootLLCI	BootULCI
-,800	-,009	,014	-,038	,021
-,109	,000	,011	-,021	,025
,951	,014	,014	-,011	,046

Index of moderated mediation:

	Index	BootSE	BootLLCI	BootULCI
C	,013	,011	-,007	,036

INDIRECT EFFECT:
T -> BIS15 -> PPU#1

C	Effect	BootSE	BootLLCI	BootULCI
-,800	,010	,015	-,015	,044
-,109	,013	,012	-,007	,043
,951	,018	,015	-,007	,051

Index of moderated mediation:

	Index	BootSE	BootLLCI	BootULCI
C	,004	,009	-,014	,024

*********************** ANALYSIS NOTES AND ERRORS ************************

Level of confidence for all confidence intervals in output:
95,0000

Number of bootstrap samples for percentile bootstrap confidence intervals:
5000

W values in conditional tables are the 16th, 50th, and 84th percentiles.

NOTE: A heteroscedasticity consistent standard error and covariance matrix estimator was used.

------ END MATRIX -----
